# Structural, ferroelectric and dielectric properties of multiferroic YMnO_3_ synthesized via microwave assisted radiant hybrid sintering

**DOI:** 10.1016/j.heliyon.2019.e01691

**Published:** 2019-05-10

**Authors:** Manish Kumar, D.M. Phase, R.J. Choudhary

**Affiliations:** a Pohang Accelerator Laboratory, POSTECH, Pohang, 790-784, South Korea; b UGC-DAE Consortium for Scientific Research, Indore, 452001, India

**Keywords:** Materials science, Condensed matter physics

## Abstract

In this work, multiferroic YMnO_3_ ceramic samples prepared via microwave assisted radiant heating method (at 4 different microwave power percentage: 0, 15, 30 and 50) were studied to investigate their structural, ferroelectric and dielectric properties. In Raman measurement, the A_1_ Raman scattering line at ∼ 683 cm^−1^ is much stronger than other Raman modes and dominates the Raman spectra in all samples. The observed Raman modes identify the hexagonal YMnO_3_ type structure of studied samples. YMnO_3_ samples prepared at different microwave power displayed variation in ferroelectric polarization. The dielectric constant and dielectric loss tangent variation across different frequencies is also explored for these samples.

## 1 Introduction

Multiferroic materials have received huge attention from the scientific community across the globe in last decade due to their potential for technological applications such as memory elements, field-effect transistors (MFIS-FET), spintronic devices, sensors etc. [1, 2, 3] Among these, YMnO_3_ (YMO) has emerged as one of the famous multiferroic material due to absence of volatile elements and room temperature ferroelectricity [4, 5, 6]. It displays high temperature ferroelectric transition at *T*_*C*_ ∼ 900K while anti-ferromagnetic transition at *T*_*N*_ ∼ 70K. Single crystal YMO is known to have dielectric constant ≈20 and remnant polarization (*Pr*) ≈ 5 μC cm^-2^ [7, 8]. YMO was studied earlier in bulk, nanomaterials, single and bilayer thin films form to achieve better functional properties for device realization and basic understanding [9, 10, 11, 12, 13]. In continuation, microwave assisted radiant hybrid sintering (MARH) method was employed to prepare multiferroic YMO samples [14]. Although their magnetic properties are reported earlier, but their ferroelectric and dielectric properties are not explored yet. Therefore, the principal objective of the present work is to investigate the structural and the ferroelectric properties of the YMO ceramics synthesized via MARH. YMO ceramic samples prepared by MARH at different microwave (MW) power percentage (0, 15, 30 and 50) were characterized by means of Raman scattering, ferroelectric and dielectric measurements to probe their structural and electrical properties.

## 2 Materials & methods

The polycrystalline samples of YMO studied in this work were prepared by standard solid state reaction route via MARH. The detailed synthesis process adopted to prepare these samples is already reported elsewhere [14]. During MARH sintering of YMO ceramic samples, different percentage of microwave power i.e. 0, 15, 30 and 50 %, henceforth the corresponding samples are designated as YMO0, YMO15, YMO30 and YMO50 respectively. Room temperature Raman measurements on synthesized samples were performed using a LABRAM HR800 spectrometer with a 632.8 nm excitation laser source equipped with a Peltier cooled CCD detector and the laser beam was focused on the sample by a 50× lens to give a spot size of 1 μm. The resolution of the Raman spectrometer is better than 2 cm^−1^. To avoid sample degradation, laser power was kept on 5 mW. Silver contacts were made on the samples for ferroelectric and dielectric measurements. Ferroelectric measurements were performed by ferroelectric loop (P-E) tracer of M/s Radiant Instruments, USA. Room temperature dielectric measurements were carried out on an impedance analyzer (Novocontrol Technologies Alpha ATB, Germany) working in the frequency range of 3 μHz–20 MHz and in the ac voltage range from 100 mV to 3 V.

## 3 Results and discussion

**Fig. 1:**
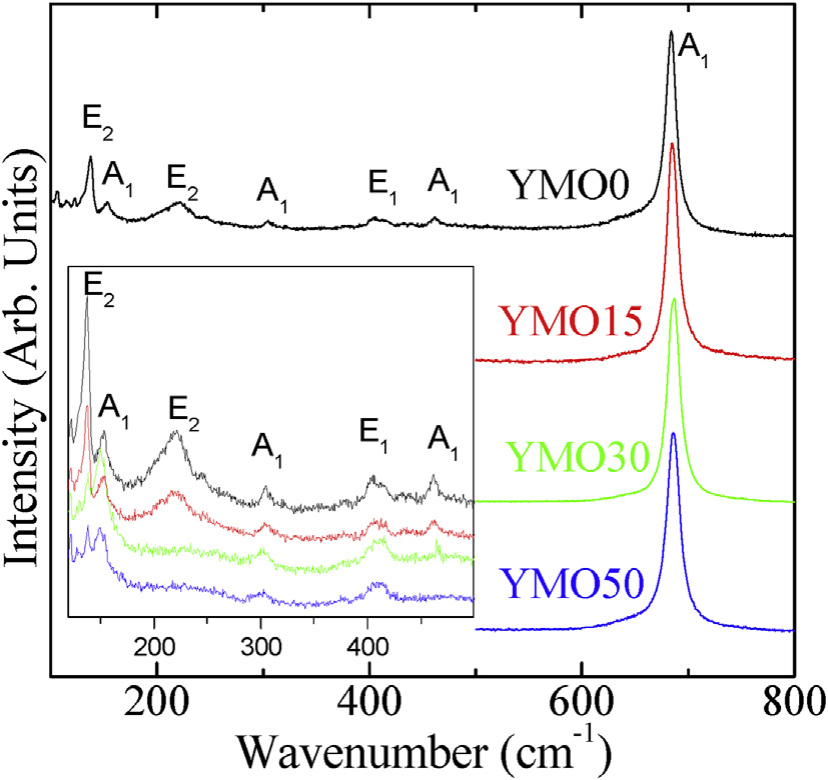
Raman spectra (measured at room temperature) of YMO ceramics prepared with different microwave powers applied during MARH. The inset depicts the region with less intense Raman modes for all samples.

Raman spectroscopy is a powerful tool for studying lattice dynamics of solids and it can provide information about subtle local variations in structures which may be hard to divulge through diffraction techniques. So, this unique vibrational technique can be employed in tandem with diffraction techniques to analyze the non-apparent local structural features, which often lead to remarkable functional properties. Fig. 1 illustrates the room temperature Raman spectra of phase pure YMO ceramic samples prepared with MARH. Interestingly, the Raman scattering line at ∼ 682 cm^−1^ is very intense in all samples and dominates the Raman spectra. In order to have more clarity on the less intense Raman modes, the insert of Fig. 1 shows Raman spectra of all samples without this intense Raman scattering mode. If we recall the crystal structure of YMO, it's hexagonal (space group *P6*_*3*_*cm*) elementary cell is composed of six formula units and for this group theory calculation predicts a total of 60 phonon modes (*Γ*_*-point modes*_ = 10*A*_*1*_+5*A*_*2*_+10*B*_*1*_+5*B*_*2*_+15*E*_*1*_+15*E*_*2*_). Among these predicted phonon modes, only 38 (*Γ*_*Raman*_ = 9*A*_*1*_+14*E*_*1*_+15*E*_*2*_) are Raman active. In this work, the observed Raman modes in YMO samples prepared with MARH are tabulated in Table 1. For YMO0 sample, the experimentally observed Raman scattering bands at 153, 304, 461, and 683 cm^−1^ are of *A*_*1*_ symmetry, those at 409 cm^−1^ are of *E*_*1*_ symmetry, and the bands at 137 and 220 cm^−1^ are of *E*_*2*_ symmetry. The observed Raman modes are in good agreement with previous studies on YMO and thus confirm the single phase growth of grown samples [15, 16, 17]. The reason behind the intense Raman mode near 683 cm^−1^ in YMO is already explained earlier [15]. This mode is related to apical oxygen atoms stretching along *c*-axis and a slight blue shift for this mode is observed in YMO samples prepared with MW. Apart from this mode, the Raman modes at 137cm^−1^ (*E*_*2*_) and 410 cm^−1^ (*E*_*1*_); appear at the same position for samples prepared with different MW percentage. *E*_*2*_ mode near 220 cm^−1^ depicts unlike behavior for different samples. This mode includes displacement of Mn and oxygen ions in the c-plane. Fukumura et al. [16] argued earlier that this mode may effectively modulate the Mn–O–Mn bond angle in the c-plane and can display anomalous behavior. It is well known that for a Raman active phonon mode, the atomic displacements modulate the macroscopic polarizability and the induced polarizability is responsible for the observed intensity of the corresponding Raman line. Moreover, the Raman mode frequencies are largely dependent on unit cell volume. In our previous study [14] on these YMO samples prepared at different MW power, we have witnessed a slight variation in the unit cell parameters obtained from the X-ray diffraction results. This slight variation in lattice parameters means a variation of particular bond length which is eventually responsible for the shift of Raman mode position. Hence, the slight variation in unit cell parameters is responsible for the shift observed in some Raman modes mainly for the YMO samples prepared at higher MW power.

**Table 1:** Raman mode positions (cm^−1^) observed at room temperature in YMO ceramic samples prepared with MARH.

Raman Modes	YMO0	YMO15	YMO30	YMO50
E_2_	137	138	138	138
A_1_	153	153	150	151
E_2_	220	220	-	-
A_1_	304	304	303	300
E_1_	409	410	410	410
A1	461	461	465	-
A1	683	685	686	686

**Fig. 2:**
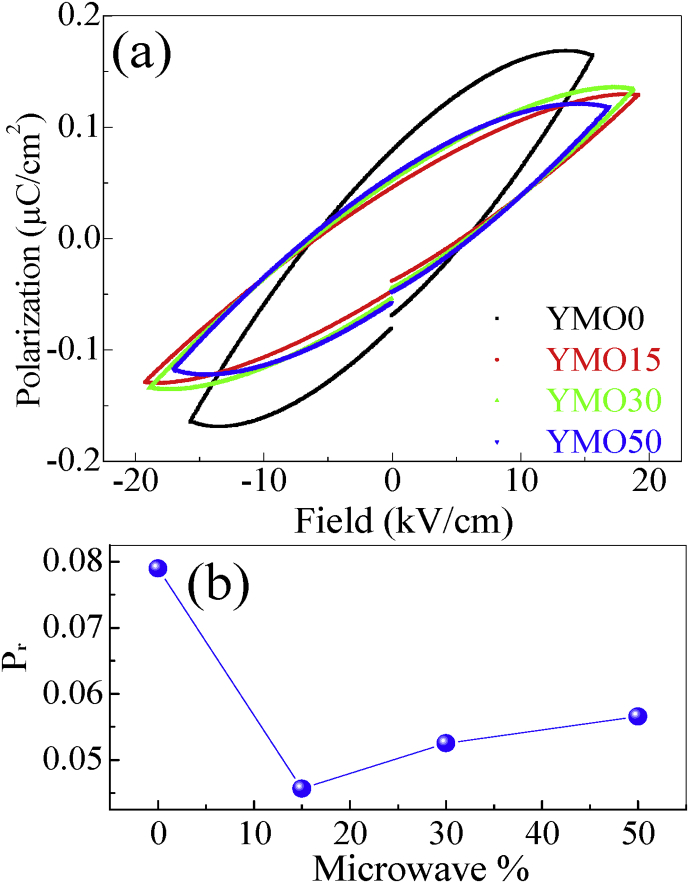
(a) Room temperature P-E data of YMO ceramic samples prepared with different microwave power applied during MARH. (b) Variation of saturation polarization (at highest measuring voltage) with the microwave power applied during MARH for studied samples.

**Fig. 3:**
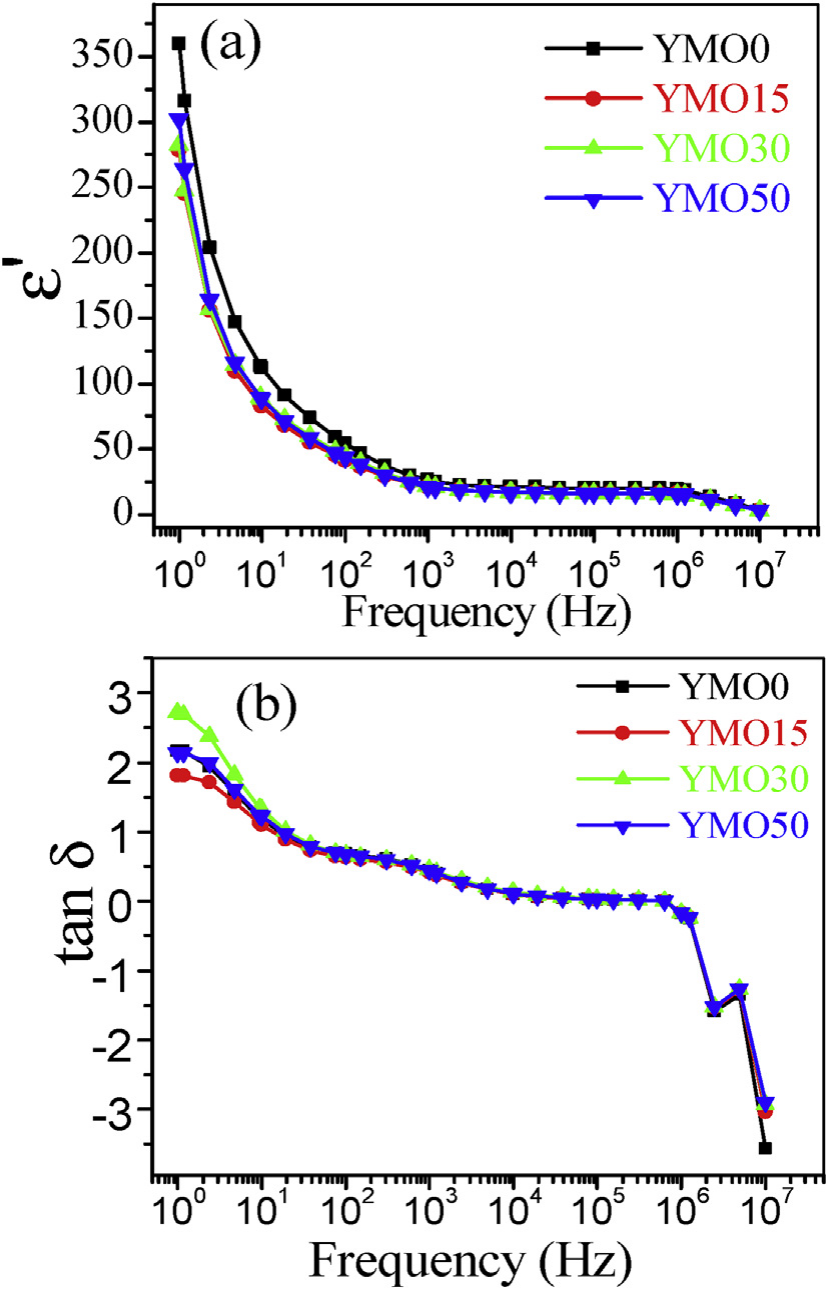
Room temperature frequency dependent (a) Dielectric constant (ε) variation and (b) Tanδ data of YMO ceramic samples prepared with MARH.

After analyzing the crystal structure, we have examined the ferroelectric properties of YMO samples synthesized via MARH. Fig. 2(a) depicts the polarization verses the electric field (P-E) plots for the studied samples measured at room temperature. The nature and magnitude of the ferroelectric loop for YMO0 sample is similar to that of YMO ceramic sample synthesized previously via an auto-combustion method [18]. Fig. 2(b) shows variation of *Pr* (at highest measuring voltage) with the microwave power applied during MARH for studied samples. Decrease in *Pr* magnitude is noticed for YMO samples prepared in presence of MW as compared to YMO sample prepared via conventional heating. This variation in Pr may be originated by the slight alteration in lattice parameters. Such deviation in lattice parameters was witnessed in these samples in x-ray diffraction measurements [14]. The frequency-dependent dielectric constant (ε′) and dielectric loss (tan δ) data of the YMO samples prepared with MARH are depicted in Fig. 3(a) and Fig. 3(b) respectively. One can note that all the studied YMO ceramic samples represent a declining tendency of ε′ with increasing frequency in the measured range 10 Hz to 10 MHz. The similar trend (with frequency) is followed by the tan δ loss curve as well. The large values of ε'and tan δ loss curves at lower frequencies can be ascribed to the space charge polarization effect [17].

There may be two possibilities for alteration of polarization and dielectric constant in MARH synthesized YMO samples as compared to YMO0 sample. First probability is the crystal structure of the phase i.e. the smaller c/a means the crystal structure is closer to the cubic structure and is likely to offer lesser ferroelectricity and dielectric constant values. In the second probability, the grain size/grain boundaries too have a substantial effect on the ferroelectric and dielectric properties. It is known that single crystal of a ferroelectric material generally comprises ferroelectric domains separated by interfaces called domain walls and the dielectric constant is dependent on the population of these domains and their mobility. The dielectric ceramic can be considered as a composite comprising ferroelectric grain cores with high dielectric constant and insulated grain boundary layers with low dielectric constant. A decrease in grain size increases the volume fraction of the grain boundary (i.e. the grain boundary density) and led to depressed dielectric tunabilities. Moreover, dielectric constant can also increase for increase in uniformity of grain size as the domain wall movement is relatively easy and regular. So, the increase or decrease in grain size as well as the uniformity in grain size are two vital parameters for the dielectric and ferroelectric properties. It needs to be recalled here that for YMO ceramic samples, MARH has resulted in uniformity of grain size plus reduction in grain size up to some extent [14]. These two types of changes in grain size affect in an opposite manner to the dielectric and ferroelectric properties and may counterbalance each other's affect. That could be the possible reason we have not observed much changes in the dielectric properties of measured YMO ceramic samples prepared via MARH.

Consistent studied were carried out in past and still underway in order to improve the functional properties of multiferroic ceramic samples and one of the focal point of these studies is their synthesis via different routes. The study on dielectric and ferroelectric properties of YMO ceramic samples synthesized via MW assisted synthesis are not reported earlier. But such studies can be vital to improve the ferroelectric and dielectric properties of such ceramic samples. In this work, we have attempted to study the ferroelectric and dielectric properties of YMO ceramic samples synthesized via MARH. We found a variation in ferroelectric properties but dielectric properties were not much affected by the MARH. Similar multiferreoic materials of this class need to be studied in this direction to reach on a general conclusion.

## 4 Conclusion

YMO ceramic samples prepared via MARH were investigated in terms of their crystal structure, ferroelectric behavior and dielectric nature. Raman analysis exhibits that all the samples have formed pure hexagonal phase and Raman scattering line at ∼ 683 cm^−1^ dominates the Raman spectra in all samples. Ferroelectric polarization in YMO samples shows dependency on MW power used during the MARH synthesis. On the other hand, it is realized that the MW power employed in MARH has less influence on the dielectric constant and dielectric loss tangent variation across different frequencies.

## Declarations

### Author contribution statement

Manish Kumar: Conceived and designed the experiments; Performed the experiments; Analyzed and interpreted the data; Contributed reagents, materials, analysis tools or data; Wrote the paper.

D. M. Phase, R. J. Choudhary: Conceived and designed the experiments; Contributed reagents, materials, analysis tools or data.

### Funding statement

This research did not receive any specific grant from funding agencies in the public, commercial, or not-for-profit sectors.

### Competing interest statement

The authors declare no conflict of interest.

### Additional information

No additional information is available for this paper.
